# Characteristics and Trends of Hospitalized Pediatric Abuse Head Trauma in Wuhan, China: 2002–2011

**DOI:** 10.3390/ijerph9114187

**Published:** 2012-11-15

**Authors:** Xin Xia, Joe Xiang, Jianbo Shao, Gary A. Smith, Chuanhua Yu, Huiping Zhu, Huiyun Xiang

**Affiliations:** 1 School of Public Health, Wuhan University, 115 Donghu Road, Wuhan, 430071, China; Email: xiaxin84@163.com (X.X.); yuchua@163.com (C.Y.); 2 Case Western Reserve University, Cleveland, OH 44120, USA; Email: yxx125@case.edu; 3 Wuhan Children’s Hospital, 100 Hongkong Road, Wuhan, 430016, China; Email: shaojb2002@sina.com; 4 Center for Injury Research and Policy, The Research Institute at Nationwide Children’s Hospital, 700 Children’s Drive, Columbus, OH 43205, USA; Email: Gary.Smith@nationwidechildrens.org; 5 The Ohio State University College of Medicine, 370 West 9th Avenue, Columbus, OH 43210, USA; 6 School of Public Health and Family Medicine, Capital Medical University, 10 Xitoutiao, Youanmen, Beijing, 100069, China

**Keywords:** abusive head trauma, child abuse, children, China

## Abstract

This study investigated characteristics and trends of hospitalized abuse-related traumatic brain injuries (TBI) treated at a large pediatric medical center in Wuhan, China during the past 10 years. De-identified hospital discharge data for patients 0–4 years old hospitalized at the Wuhan Medical Care Center for Women and Children were analyzed, and ICD-10 codes were used to identify cases of TBI. Medical notes provided by doctors in the medical record were used to identify TBI cases in which suspected child abuse was the cause. From 2002 to 2011, 3,061 pediatric TBI patients were hospitalized and 4.6% (140) of these cases were suspected child abuse-related. The majority of suspected child abuse cases involved children younger than 1 year of age (68.6%) and usually affected males (63.6%). Children with non-Abusive Head Trauma (AHT) were more likely to have full recovery outcome (68.4%, 95% CI: 66.6%–70.0%) than children with suspected AHT (44.3%, 95% CI: 36.1%–52.5%). The proportion of all childhood TBI attributable to abuse did not appear to have increased in the 10-year period at this medical center. This is the first comprehensive study highlighting the important role of suspected child abuse in causing TBIs among Chinese children. Child abuse as a major cause of TBIs among infants in China should be studied further, and there should be greater awareness of this important social and medical problem in China.

## 1. Introduction

Child abuse, a common and serious problem around the World, refers to all forms of neglect, physical, emotional or sexual abuse that results in harm to a child’s health, development or dignity [1,2,3]. Every year, 155,000 deaths occur in children globally as a result of abuse or neglect [4], yet the number of abused children has been and continues to be largely underestimated [5]. The incidence of child abuse in the USA is estimated at 23.1 per 1,000 children [5], and an estimated 100,000–160,000 children are victims of child abuse annually in The Netherlands [6]. Children who suffered child abuse can develop physical, emotional, and cognitive conditions, which can significantly impact neurological development [7]. As such, prevention of child abuse and the reduction of abusive injuries and their negative impacts should be a global health priority.

The leading cause of child abuse fatalities is abusive traumatic brain injury (TBI), which includes shaken baby syndrome (SBS) and other abuse-related head trauma [8,9,10]. Abusive head trauma (AHT) may lead to significant morbidity among survivors，and is remarkably over-represented for children in the first year of life [11]. The incidence of AHT ranges from 20 to 30 cases per 100,000 children under 1 year of age, with the peak incidence at 3 months of age [12,13,14,15,16]. Previous studies indicate that children with AHT appear to have more severe consequences than children with similar severity, non-abusive injuries [17,18]. Approximately 7–30% of infants with AHT die, 30–50% experience significant neurological and cognitive deficits, and up to 30% have long-term adverse effects [19,20].

In China, social and cultural traditions as well as a lack of public awareness regarding appropriate child discipline has caused child abuse to remain as an undetected issue. Most Chinese people still follow a deep-rooted traditional belief in “spare the rod, spoil the child.” As a result, a large proportion of Chinese people have experienced some types of physical abuse during their childhood [21]. Researchers found that although Chinese children are diagnosed with fractures, disability, and even death because they are struck by their parents, there are still a certain percentage of parents who do not realize that their behaviors constitute child abuse, and instead believe that their type of discipline is the normal way of educating their children [22]. Furthermore, child abuse remains undetected in China because the healthcare system lacks a standardized procedure in reporting child abuse cases. Not only is there no official standard in defining child abuse, but physicians are also not mandated by Chinese law to report child abuse or suspected child abuse. It should also be noted that most physicians in Chinese hospitals lack training and expertise in identifying child abuse cases. As such, it is of utmost importance to raise awareness about abusive head trauma among pediatricians and policymakers, as well as among the general public in China.

Research about epidemiological characteristics and outcomes of TBI were mostly conducted in adult Chinese population. An epidemiologic study investigating 246,812 participants living in rural and urban areas from 21 provinces of China found that assaults were the third leading causes of TBI in both areas [23]. However, evidence suggests that data about abusive TBI among children are unavailable in China, neither from official registries nor in scientific literatures. Taking into consideration the large information gap about pediatric AHT in China, as well as the significant adverse effects of AHT, this study was designed to investigate epidemiological characteristics of hospitalized children with suspected AHT from 2002 to 2011 at a large urban pediatric medical center in China. Overall, this study hypothesized that: (1) the majority of hospitalizations for AHT would occur in children less than 2 years of age, and (2) girls were more likely to be abused than boys among AHT cases. This study also investigated the trend of proportion (%) of suspected pediatric abusive head trauma (AHT) in all inpatient children 0–4 years of age with traumatic brain injuries from 2002 to 2011, a period that saw rapid economic developments and dramatic social changes in China.

## 2. Methods

### 2.1. Setting

Wuhan Medical Care Center for Women and Children (WMCCWC) is the largest regional hospital providing clinical services and health promotion and prevention for women and children in central China. WMCCWC has four outpatient departments, two inpatient buildings and 1,112 beds serving Hubei and the surrounding provinces. Each year, approximately 30,000 inpatients and a total of 900,000 children are treated at outpatient and emergency departments (ED) of WMCCWC. Data from the Wuhan Bureau of Health indicated that about 55%~60% of total pediatric outpatients and ED patients in Wuhan city were treated at the WMCCWC. De-identified hospital discharge data from the WMCCWC were analyzed for this study. This study was approved by the institutional review board of Tongji Medical College School of Public Health in Wuhan, China.

### 2.2. Subjects

The study population consisted of children less than 5 years of age admitted to WMCCWC with a discharge diagnosis of traumatic brain injury from January 1, 2002 to December 31, 2011. Data were retrospectively collected via reviewing the medical records database of the WMCCWC. Subjects were classified as “suspicious for abuse” and “non-abuse” according to the notes in the medical record written by physicians who treated the patient. In WMCCWC, for the diagnosis of suspected AHT, physicians mainly rely on the combination of radiographic and physical examination information, and history of the injury provided by the caretaker. Every child attending to the WMCCWC needs to be evaluated by a doctor. When the doctor finds some physical symptoms of inflicted injuries (e.g., whip scars, occult multiple fractures, bruises, subcutaneous bleeding or hematoma) in combination with a head injury, he/she may ask the adult caregiver about the detailed injury history. If there was a witnessed inflicted head injury or confession of intentional injury by a caregiver, then the doctor make notes in the medical record. In China, making such notes is still a voluntary behavior of doctors. In this study, each medical record was reviewed by one of the authors (Xin Xia at the Wuhan University School of Public Health) who categorized patients into two groups mentioned above. Subjects were excluded from the study if there was not enough information in the medical record to determine if the injury was “suspicious for abuse” and “non-abuse”. Unlike the USA and other countries, physicians in China are not required to confirm child abuse cases or refer them to a child protection agency. As such, this study could only use “suspected child abuse” herein to report these cases, since a systematic legal procedure for confirming suspected child abuse cases does not exist in China.

### 2.3. Measures

Data were extracted from the WMCCWC medical records database for all eligible subjects. Diagnosis codes of all inpatients treated at the WMCCWC were assigned using ICD-10 codes. Information collected from the medical record included: patient identification number, date of birth, gender, date of admission, admission diagnosis, severity of condition at admission, head CT scanning results, discharge date, discharge ICD-10 codes, type of conditions (injury and poisoning or other), description of injury events and stated cause of injury, outcomes at discharge, and length of stay. The stated cause of injury was defined as the injury mechanism reported by the caretakers when the child was admitted to the hospital. Causes of injury were classified as suspected child abuse or non-abuse (traffic crashes, falls, strike by/against objects, sports, and other causes) in this study.

### 2.4. TBI Case Definition

TBI cases in the WMCCWC medical record were identified using the USA definition of TBI based on the International Classification of Diseases, Tenth Revision (ICD-10) diagnosis codes. These codes included S01.1-S01.9, S02.0-S02.3, S02.7-S02.9, S04.0, S06.0-S06.9, S09.7-S09.9, T90.1, T90.2, T90.4, T90.5, T90.8, and T90.9. A total of 3,061 hospitalized TBI cases among children aged 0–4 years were identified. All suspected child abuse cases were required to have detailed and complete documentation of the injury circumstances either from an eyewitness, a caregiver, or any other person involved who admitted an abusive behavior that caused the TBI. This information was provided by the physician who treated the child and wrote notes about suspected abuse in the patient medical record.

### 2.5. Data Analysis

Suspected AHT and non-AHT were compared with respect to gender, age, outcome, and length of hospital stay using Chi-squared test and student *t *test. Frequency and percentage distribution of suspected AHT and non-AHT hospitalizations by age were calculated along with the proportion of suspected AHT among all TBI cases. Age distribution (in months) of suspected AHT and non-AHT in children <2 years old was also provided. Proportions (%) and 95% confidence intervals (95% CI) of suspected AHT in all inpatient children with TBI for each year from 2002–2011 were calculated. All statistical analyses were performed using the SAS statistical software. Any *p*-value less than or equal to 0.05 was considered statistically significant.

## 3. Results

Among 3,061 inpatient children with traumatic brain injuries, 140 were identified as suspected AHT cases, and 63.6% of AHT cases were boys (Table 1). There was no significant gender difference between children with suspected AHT and non-AHT; however two groups differed significantly with regard to age distribution. The age groups of children who had the highest proportion of AHT were *<*1 year old (96, 68.6%), 1 year old (14, 10.0%), and 2 year olds (16, 11.4%). Among all inpatients with suspected AHT, 68.6% occurred among children <1 year of age compared with 23.2% of non-AHT inpatients. The proportion of TBI caused by suspected child abuse in all pediatric TBI decreased from 12.4% in children <1 year to 1.2% in children 4 years of age (data not shown). The mean length of hospital stay was 7.8 days for children with suspected AHT and 7.0 days for children with non-AHT (*p* = 0.060, *t *test). When discharged from the hospital, children with non-AHT were more likely to have full recovery outcome (68.4%, 95% CI: 66.6%–70.0%) than children with suspected AHT (44.3%, 95% CI: 36.1%–52.5%).

**Table 1:** Characteristics of hospitalized children with suspected AHT (n = 140) and non-AHT (n = 2,921).

	Suspected AHT		Non-AHT		*p* Value
	Sample N	% (95% CI)	Sample N	% (95% CI)	
**Gender**					0.868
Male	89	63.6 (55.6–71.5)	1,877	64.3 (62.5–66.0)	
Female	51	36.4 (28.5–44.4)	1,044	35.7 (34.0–37.5)	
**Age (Years)**					<0.001
<1	96	68.6 (60.9–76.3)	680	23.2 (21.8–24.8)	
1	14	10.0 (5.0–15.0)	530	18.1 (16.8–19.5)	
2	16	11.4 (6.16–16.7)	781	26.7 (25.1–28.3)	
3	9	6.4 (2.4–10.5)	528	18.1 (16.7–19.5)	
4	5	3.6 (0.5–6.7)	402	13.8 (12.5–15.0)	
**Outcomes**					<0.001
Full recovery	62	44.3 (36.1–52.5)	1,994	68.4 (66.6–70.0)	
Partial recovery	77	55.0 (46.7–63.2)	842	28.5 (27.2–30.5)	
Death	0	0	8	0.3 (0.1–0.5)	
Unknown	1	0.7 (0.0–2.1)	77	2.8 (2.1–3.2)	
**Length of stay (days)**					
Mean (S.D.)	140	7.8 (4.5)	2,921	7.0 (5.9)	0.060
Min-Max		1.1–23.6		0.3–166.9	

**Figure 1:**
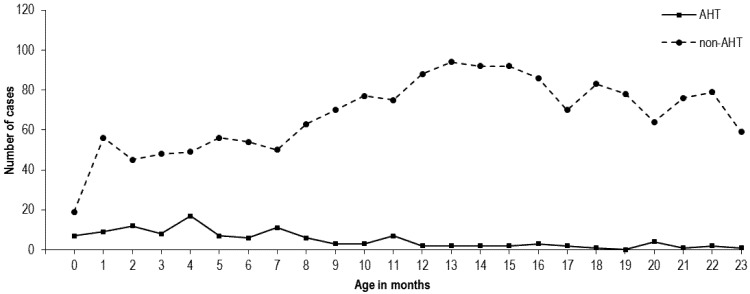
Number of hospitalized children <2 years with suspected AHT and non-AHT by month of age.

Figure 1 shows the number of hospitalized head injury cases by months of age in children 2 years old or younger, for both suspected AHT and non-AHT patients. The peak of age in AHT group was 4 months. In contrast, the non-AHT group illustrated a peak at 13 months of age.

**Figure 2:**
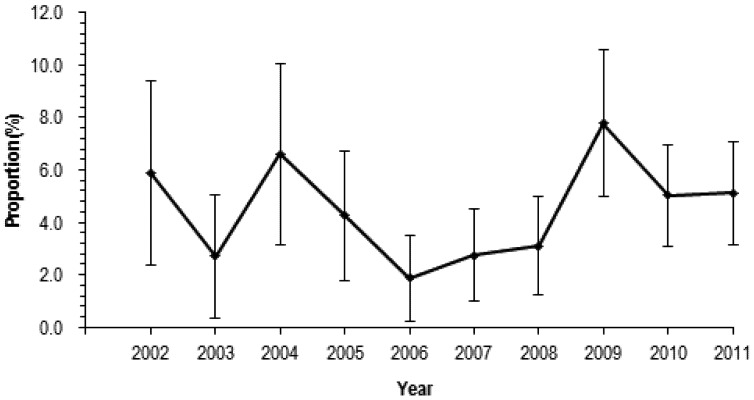
Year trend of proportion of suspected AHT in all inpatient children 0–4 years of age with traumatic brain injuries.

Figure 2 reports the year trend of proportion (%) of suspected AHT in all inpatient children 0–4 years of age with traumatic brain injuries from 2002 to 2011. The proportion of TBI caused by suspected child abuse was at the lowest level of 1.9% (95% CI = 0.3%–3.5%) in 2006. After 2006, proportion of TBI caused by suspected child abuse increased and peaked in 2009 (7.8%, 95%CI = 5.0%–10.6%).The proportion of all childhood TBI attributable to abuse did not appear to have increased in the 10-year period.

## 4. Discussion and Study Limitations

Previous studies about abusive head trauma in children were mostly from high income countries [24,25,26,27]. Our study is possibly the first study that reports the characteristics of abuse-related TBIs among children in China. We found that 63.6% of AHT patients were boys. A similar finding was reported in previous studies: 60.0% of boys in a study of AHT using the Healthcare Cost and Utilization Project (HCUP)-Nationwide Inpatient Sample (NIS) database; 67.2% of boys in a study of North Carolina traumatic brain injury registry data; and 57.2% of boys in a North Carolina review of hospital medical records [28,29,30].

Interestingly, we did not find a statistically significant difference between boys (4.5%, 89/1,966) and girls (4.7%, 51/1,095) in the proportion of suspected AHT in all pediatric head trauma patients (*p* = 0.868). This finding did not support our initial hypothesis that girls were more likely to experience abuse than boys among all children with TBI. Traditionally, the idea that men are superior to women exists in China, so parents may treat boys more favorably and are less likely to abuse boys than girls. Our result based on pediatric patients treated at this large urban medical center did not support the hypothesis that girls were more likely than boys to sustain abuse head trauma. A study of suspected AHT in pediatric head trauma patients in rural areas is warranted to further test our study hypothesis because many traditional Chinese views are still deep rooted in rural areas of China.

Our study is consistent with other existing literatures that reported that most hospitalizations for AHT occur in children <2 years of age [28,31]. Our study also found that the peak of age in children with AHT was at 4 months, which was within the range reported in several other studies (the peak hospitalization rates for AHT occurred at the first 2–4 months of life) [26,31,32,33]. Previous studies indicated that significant social stigma might have biased people’s answers and people are more likely to lie or not admitting their behaviors, especially those behaviors that are less socially acceptable [34]. This may partly interpret the relatively small number of AHT cases identified over a 10-year period in our study. In China, although child abuse is not included in China’s criminal law at present, there are still other laws and regulations like Law of the People’s Republic of China on the Protection of Minors that stipulate that the minors should not be maltreated or forsaken. However, unlike the child abuse reporting procedures in medical practice that required by laws in the USA, there is no law or regulation in China that mandates reporting of child abuse by physicians to law enforcement agencies. Therefore, underreporting of child abuse might have biased our findings in this study.

In this study, we found that children with non-AHT were more likely to have full recovery outcome at discharge than children with suspected AHT. This result was consistent with the findings from previous studies that reported worse outcomes in AHT children compared with children with non-abusive traumatic brain injury [14,35]. There are two possible explanations for this phenomenon. First, in our sample of head trauma patients, the majority of children with suspected abusive TBI were less than 1 year of age. A previous study found that younger children are much more vulnerable to sustain a head trauma than older children, and often have more severe traumatic brain injury [36]. Second, children with abusive TBI are often not brought to hospital until they have severe serious symptoms due to stigma and the caregiver’s fear of possible legal trouble [26]. Therefore, pediatric patients with AHT are significantly less likely than non-AHT children to have a good outcome at hospital discharge.

There are several limitations in our study. First, our results may not reflect the true number of AHT cases in the community, as our study subjects were selected from the medical record database in only one large regional children’s medical center in China. However, results regarding demographical characteristics, statistical comparisons, proportion, and year trend were still valid and provided preliminary findings about a significant social and medical issue in China. Second, patients in this study were retrospectively identified from the inpatient medical record database. Emergency department patients were not included because hospitals in China do not keep medical records for patients who are treated and discharged from the emergency department. Third, this study relied heavily on documentation of trauma history of the hospital patients and voluntary reporting of physicians in the medical records to identify suspicious AHT. Thus, our results might be subject to reporting bias as AHT cases were determined by a suspected child abuse note written by the physician in the medical record. In Chinese culture, some types of discipline by parents, which may be considered child abuse in Western cultures, are often believed by the Chinese society as an acceptable form of authoritative punishment. Also, physicians in China have inadequate knowledge and training to recognize abuse injury; therefore, TBI caused by child abuse might have been underreported in our study. Furthermore, there are no standard criteria for diagnosis of AHT, and previous studies in the USA used different operational definitions of AHT. Some investigators defined pediatric AHT based on the findings by a social service agency or a hospital child protective expert panel, while others used clinical criteria [37,38,39]. Finally, we could not calculate rates nor could age standardized rates be calculated to assess the year trend of suspected AHT due to lack of an appropriate denominator.

Unfortunately, there are neither special social service agencies nor a specialized evaluation team in hospitals for identifying and reporting child abuse in China. Therefore, establishing a clear case definition that allows for a more accurate assessment of the burden of abuse-related head trauma in children and evaluation of the effect of future prevention and education programs is a top priority in TBI research and prevention in China.

## 5. Conclusions

Our study confirmed the important role of suspected child abuse in causing TBIs in young children in China. This study is significant because previous publications from China have not studied children with suspected AHT. Our study provided some important preliminary findings about this social and medical problem in China.
